# Supplementary material for: A bacterial kinase phosphorylates OSK1 to suppress stomatal immunity in rice
Source: Nat Commun. 2021 Sep 16;12:5479. doi: 10.1038/s41467-021-25748-4 (PMC8445998; doi:10.1038/s41467-021-25748-4)
Supplement: Supplementary file 1 — Supplementary information [file 41467_2021_25748_MOESM1_ESM.pdf]

A bacterial kinase phosphorylates OSK1 to suppress stomatal immunity in rice

Shanzhi Wang[1], Shuai Li[1], Jiyang Wang[1], Qian Li[1], Xiu-Fang Xin[2,3], Shuang Zhou[1], Yanping Wang[1], Dayong Li[4], Jiaqing Xu[1], Zhao-Qing Luo[5], Sheng Yang He[2,6], Wenxian Sun[1,4,*]

[1]Department of Plant Pathology, the Ministry of Agriculture Key Laboratory of Pest Monitoring and Green Management, and Joint Laboratory for International Cooperation in Crop Molecular Breeding, Ministry of Education, China Agricultural University, Beijing, China

[2]DOE Plant Research Laboratory, Michigan State University, East Lansing, Michigan, USA

[3]National key Laboratory of Plant Molecular Genetics, CAS Center for Excellence in Molecular Plant Sciences, Institute of Plant Physiology and Ecology, Chinese Academy of Sciences (CAS), CAS John Innes Centre of Excellence for Plant and Microbial Sciences (CEPAMS), Shanghai, China.

[4]College of Plant Protection, Jilin Agricultural University, Changchun, Jilin, China

[5]Purdue Institute for Inflammation, Immunology and Infectious Disease and Department of Biological Sciences, Purdue University, West Lafayette, IN, USA

[6]Howard Hughes Medical Institute, Michigan State University, East Lansing, Michigan, USA

[*]Address for correspondence and proofs: E-mail: wxs@cau.edu.cn

Supplementary Information

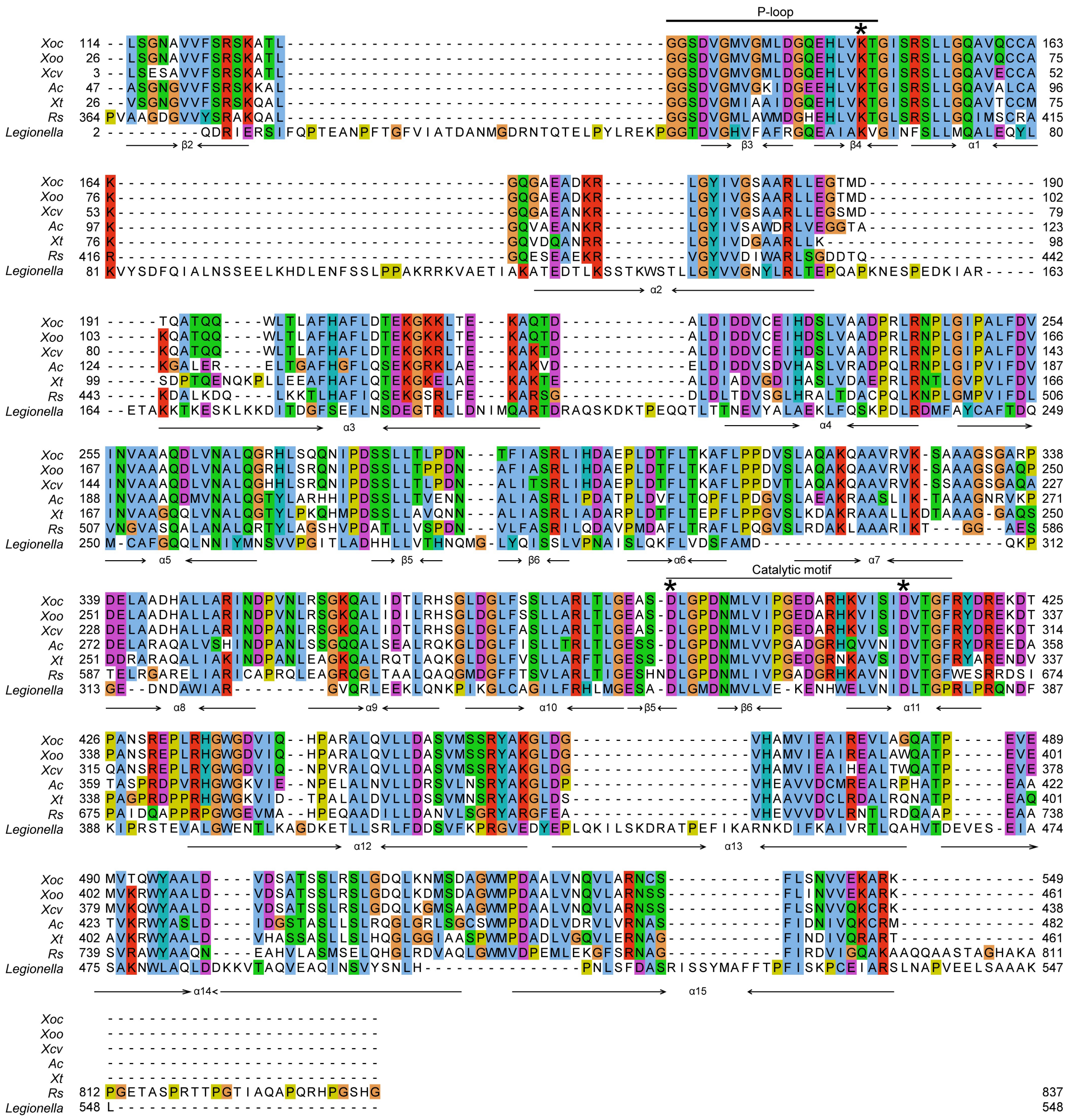

Supplementary Figure 1. Sequence alignment of XopC2 and its homologs. XopC2 homologs were identified by PSI-BLAST searches against full-length protein sequence of XopC2. The protein sequences of XopC2 and its homologs from *X. oryzae* pv. *oryzae* (*Xoo*), *X. campestris* pv. *vesicatoria* (*Xcv*), *Acidovorax citrulli* (*Ac*), *X. translucens* (*Xt*), *Ralstonia solanacearum* (*Rs*) and *Legionella shakespearei* (*Ls*), a closely relative of the human pathogen *L. pneumophila*, were aligned with COBALT, and were then colored using

JalView. The putative catalytic domain and P-loop-like motif are highly conserved and labeled. * indicates three conserved residues of the putative catalytic triad. The predicted secondary structures including α-helices and β-sheets are also marked.

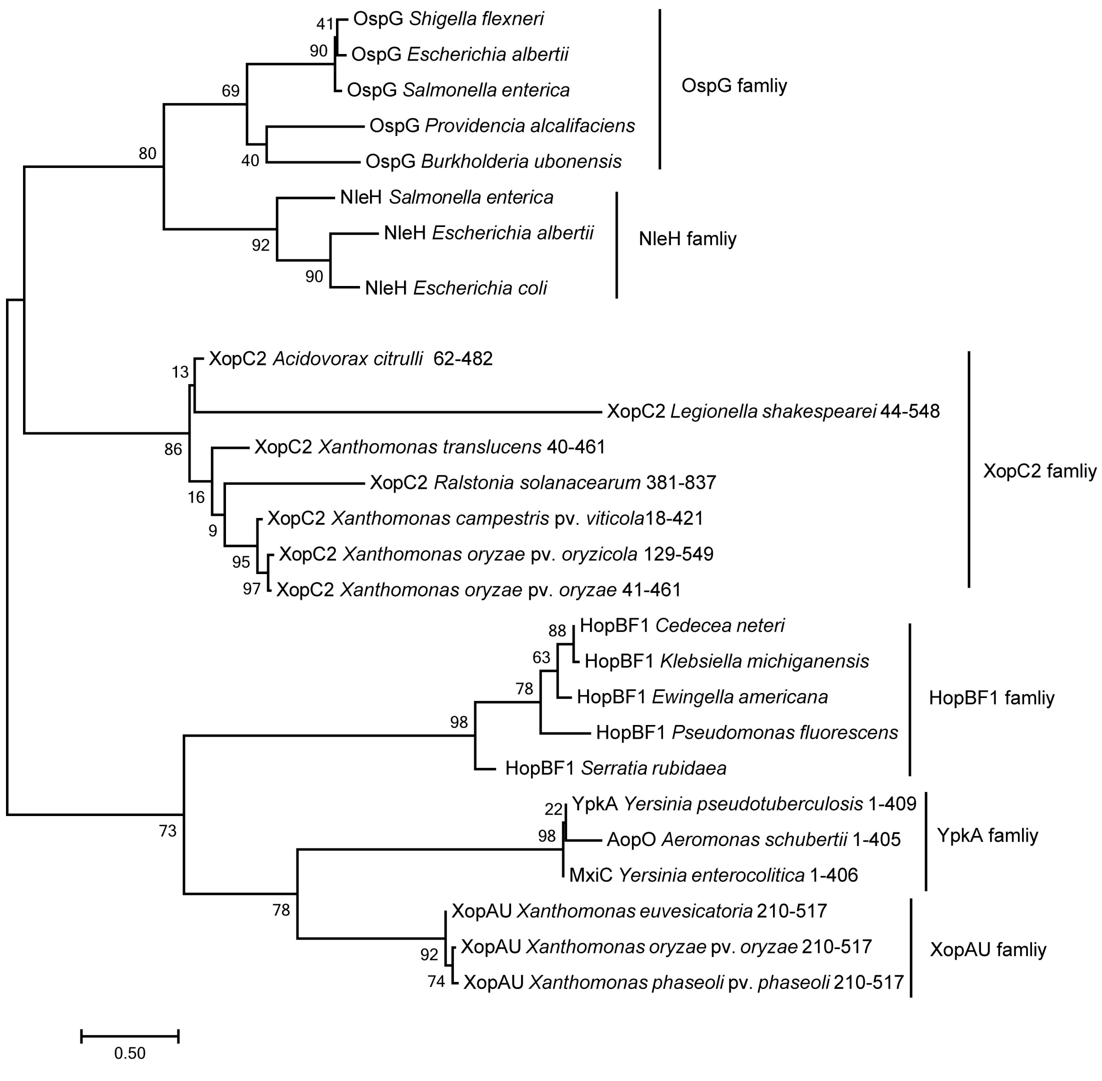

Supplementary Figure 2. Phylogenetic analysis of different families of bacterial effector kinase domains. Amino-acid sequences of different effector kinase domains were aligned with COBALT, and phylogenetic tree was constructed via MEGA 7 using the Maximum Likelihood method based on the JTT matrix-based model with 500 bootstraps.

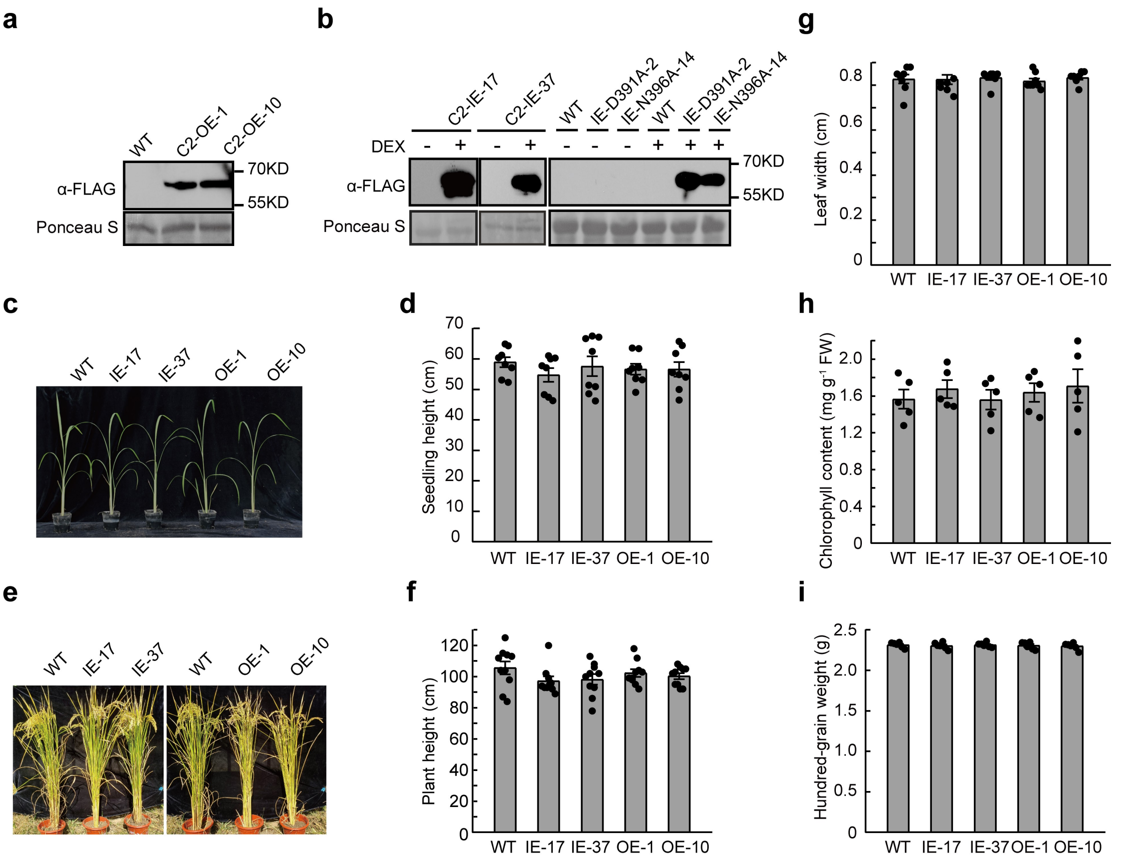

Supplementary Figure 3. The growth phenotypes and agronomic traits of the transgenic rice lines expressing XopC2 and its variants. a, The expression of XopC2-FLAG under the 35S promoter in the OE-1 and OE-10 transgenic rice lines was detected by immunoblotting. **b,** DEX-induced expression of XopC2-FLAG, XopC2^{D391A}-FLAG and XopC2^{N396A}-FLAG was detected by immunoblotting in the IE-17, IE-37, IE-D391A-2 and IE-N396A-14 transgenic rice lines. The IE transgenic lines transformed with pTA7001-*xopC2-FLAG*, pTA7001-*xopC2^{D391A}-FLAG* and pTA7001-*xopC2^{N396A}-FLAG* were treated with DEX (30 μM in 0.01% Silwet L77) or 0.01% Silwet L77 (Mock) before protein extraction. Upper panels, XopC2-FLAG and its variants were detected by western blotting with an anti-FLAG antibody in the indicated transgenic lines. Lower panels, the same blots were stained with Ponceau S to show protein loading. WT, the wild-type

plant; α-FLAG, anti-FLAG antibody; DEX, dexamethasone; -, without DEX treatment; +, with DEX treatment. **c-d,** The representative image (**c**) and the average height (**d**) of 4-week-old seedlings of the wild-type, IE-17, IE-37, OE-1 and OE-10 transgenic lines. **e**-**f**, The representative image (**e**) and the average height (**f**) of 4-month-old plants of the wild-type, IE-17, IE-37, OE-1 and OE-10 transgenic lines. **g-h**, The leaf width (**g**) and chlorophyll content (**h**) of 4-week-old seedlings of the wild-type, IE-17, IE-37, OE-1 and OE-10 transgenic lines. Leaf width was measured in the middle region of the 4th leaves for each plant. Chlorophyll was extracted from the 4th leaves with acetone. Total chlorophyll contents were measured by a spectrophotometer and calculated using the equation Chlorophyll = $20.2 \times OD_{645} + 8.02 \times OD_{663}$. **i**, The hundred-grain weight of the wild-type, IE-17, IE-37, OE-1 and OE-10 transgenic lines. Bars indicate means ± SE in **d**, **f**, **g**, **h** and **i** (n = 8, 10, 8, 5) and 6 technical replicates, respectively). These experiments were all independently repeated for 3 times with similar results. Statistical analyses showed no significant difference in agronomic traits among the wild-type and different transgenic lines in **d, f**-**i** (One-way ANOVA, Tukey's honest significance test).

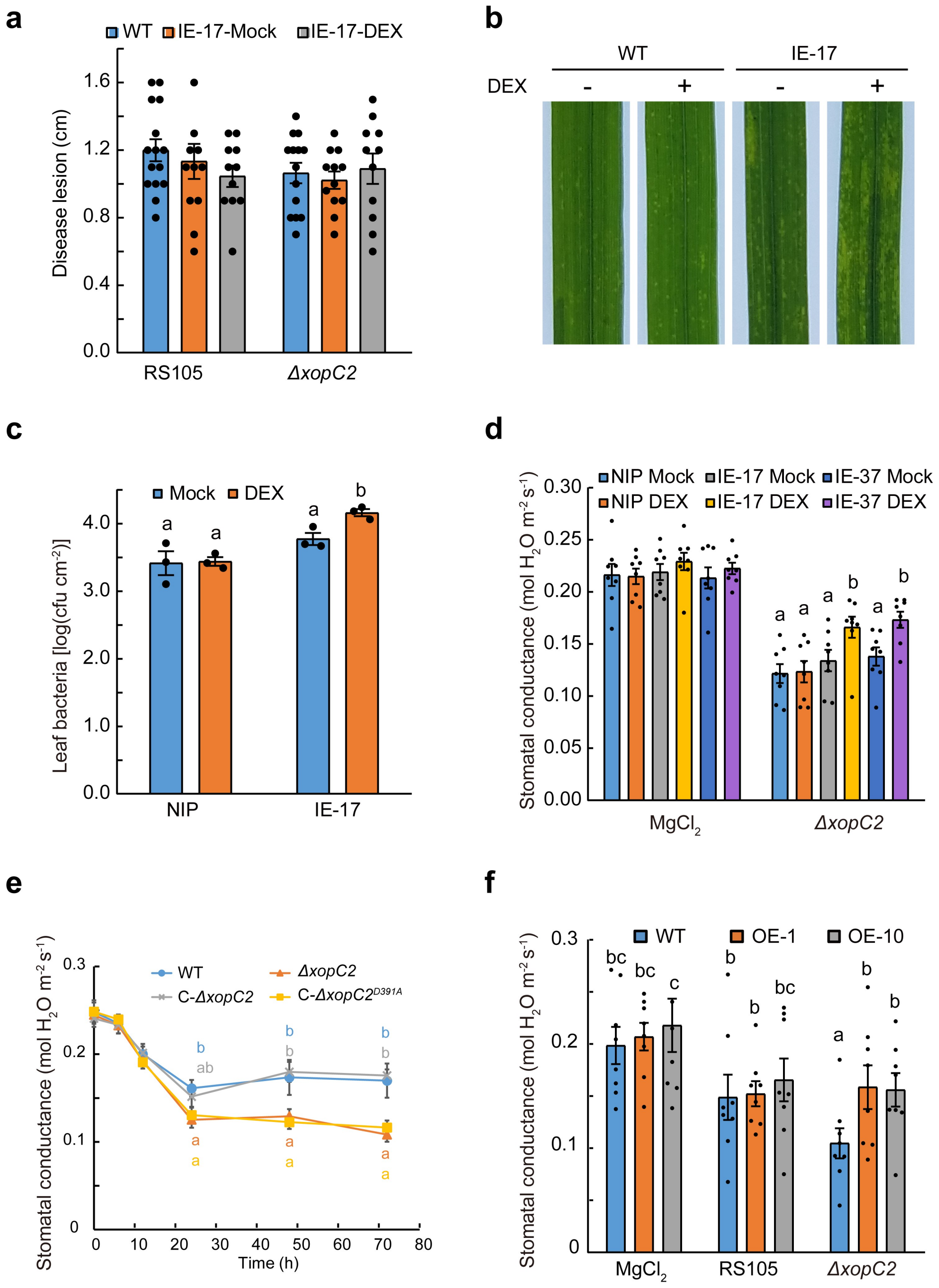

Supplementary Figure 4. Effects of XopC2 and its variants on disease symptoms and stomatal conductance in the transgenic plants after spray and pressure inoculation. a, The length of disease lesions on the pressure-inoculated wild-type (NIP) and IE-17 transgenic rice leaves after mock and DEX treatments. The wild-type and transgenic rice leaves were

pressure infiltrated with the *Xoc* RS105 and *xopC2*-knockout (*ΔxopC2*) strains at 24 hours after spraying with DEX and mock buffer. The lesion length was measured for leaves at 14 days post inoculation (dpi) (n = 15 for RS105-inoculated wild-type plant leaves, 14 for *ΔxopC2*-inoculated wild-type plant leaves, 12 for RS105-inoculated and *ΔxopC2*-inoculated mock-treated IE-17 leaves, 11 for RS105-inoculated and *ΔxopC2*-inoculated DEX-treated IE-17 leaves, respectively). Statistical analyses showed no significant difference in the length of disease lesions on the wild-type and transgenic plant leaves after pressure inoculation. **b,** Disease lesions on the *ΔxopC2*-inoculated wild-type and IE-17 transgenic plant leaves after spray inoculation. Three-week-old seedlings were sprayed with 30 μM DEX in 0.01% Silwet L77 or 0.01% Silwet L77 (Mock) followed by spray inoculation of *ΔxopC2* suspension after 24 hours. Photographs were taken at 4 dpi. **c**, Bacterial population sizes in the *ΔxopC2*-inoculated leaves of the wild-type and IE-17 transgenic plants. The 3-week-old seedlings were treated with DEX and mock buffer for 24 hours followed by spraying with *ΔxopC2*. Bacterial population was determined at 4 dpi. **d**, Stomatal conductance (Gs) of rice leaves in the wild-type, IE-17 and IE-37 transgenic lines with or without *ΔxopC2* inoculation. The DEX- and mock-treated 3-week-old seedlings were spray-inoculated with *ΔxopC2* and mock buffer. Gs was measured at 2 dpi. **e**, Time course assay on Gs of the wild-type rice leaves after challenging with RS105, *ΔxopC2*, C-*ΔxopC2* and C-$\Delta xopC2^{D391A}$. Gs was measured at 0, 6, 12, 24, 48 and 72 hpi. **f,** Gs in the wild-type and XopC2-overexpressing transgenic plant leaves after spray inoculation with the wild-type and *ΔxopC2* strains. Three-week-old seedlings were sprayed with RS105 and *ΔxopC2* suspensions. Gs was measured at 2 dpi. Data are shown as means ± SE (n = 3 technical replicates in **c** and n = 8 technical replicates in **d, e, f**). These experiments were all independently repeated for 3 times with similar results. Different letters (a-c) indicate statistically significant differences in **c**, **d, e** and **f**, as revealed by one-way ANOVA, Tukey's honest significance test.

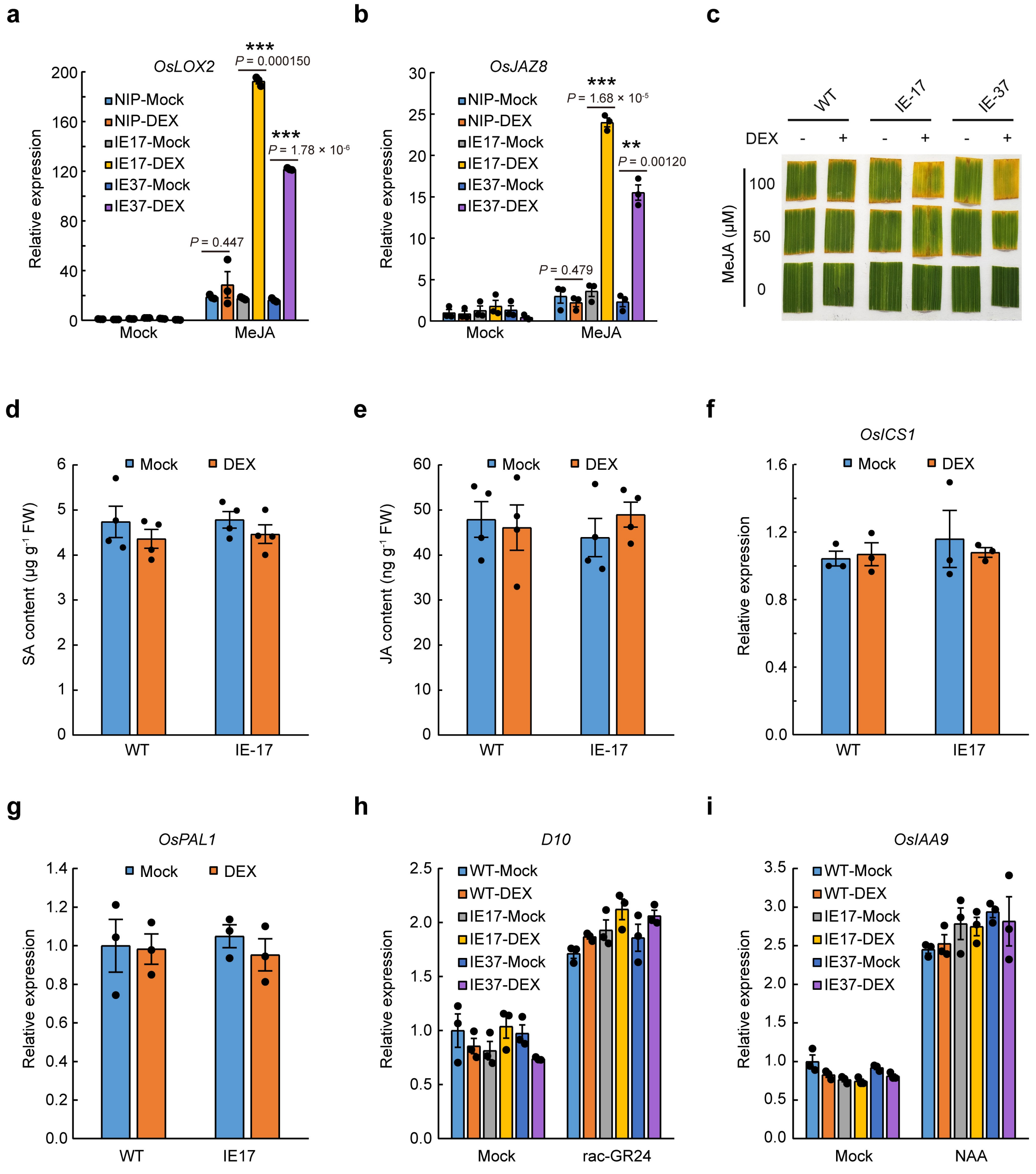

Supplementary Figure 5. Leaf senescence, jasmonic acid and salicyclic acid contents, and expression of the marker genes in jasmonate, strigolactone and auxin signaling in XopC2-expressing transgenic rice seedlings. a-b, MeJA-induced expression of the JA-responsive genes *OsLOX2* (**a**) and *OsJAZ8* (**b**) was enhanced in XopC2-expressing rice seedlings. The six-day-old seedlings were treated with DEX or mock buffer for 24 h followed by MeJA application (50 μM). Gene expression was detected by

qRT-PCR using *OsActin* as an internal reference gene. Data are presented as means ± SE (n = 3 technical replicates). * indicates a significant difference in relative gene expression levels between mock and DEX treatments (two-sided *t*-test; **, $P < 0.01$; ***, $P < 0.001$). **c,** MeJA-induced leaf senescence in the wild-type and XopC2-expressing transgenic lines. Leaf pieces were collected from the 5th leaves of DEX- and mock-treated rice plants (6-week-old) and were then incubated in 0, 50, 100 μM of MeJA for 4 days in the dark. **d-e**, The endogenous contents of SA (**d**) and JA (**e**) in the wild-type and IE-17 transgenic lines. SA and JA were extracted with acetone from the DEX- and mock-treated rice seedlings (4-week-old) at 2 days after treatment, and were quantified using ELISA. The JA and SA contents were determined by comparing the absorbance of samples to standard curve. Data are presented as means ± SE (n = 4 technical replicates). No significant difference in relative SA and JA contents was detected in the IE-17 transgenic lines after mock and DEX treatments by one-way ANOVA, Tukey's honest significance test. **f**-**g**, The expression of SA-biosynthesis genes *OsICS1* (**f**) and *OsPAL1* (**g**) in the wild-type and IE-17 transgenic seedlings at 24 hours after treatments. **h**-**i,** rac-GR24-induced expression of the strigolactone-responsive gene *D10* (**h**) or NAA-induced expression of the auxin-responsive gene *OsIAA9* (**i**) was not altered by DEX treatment in IE-17 and IE-37 transgenic rice seedlings. These seedlings (six-day-old) were treated with DEX or mock solution for 24 hours, followed by the application of 30 μM rac-GR24 or 10 μM NAA for 6 hours. Gene expression was detected as described in **a-b**. In **f**-**i**, Data are presented as means ± SE (n = 3 technical replicates). No significant difference in relative gene expression levels was detected in the IE transgenic lines after mock and DEX treatments by one-way ANOVA, Tukey's honest significance test. These experiments were all independently repeated for 3 times with similar results.

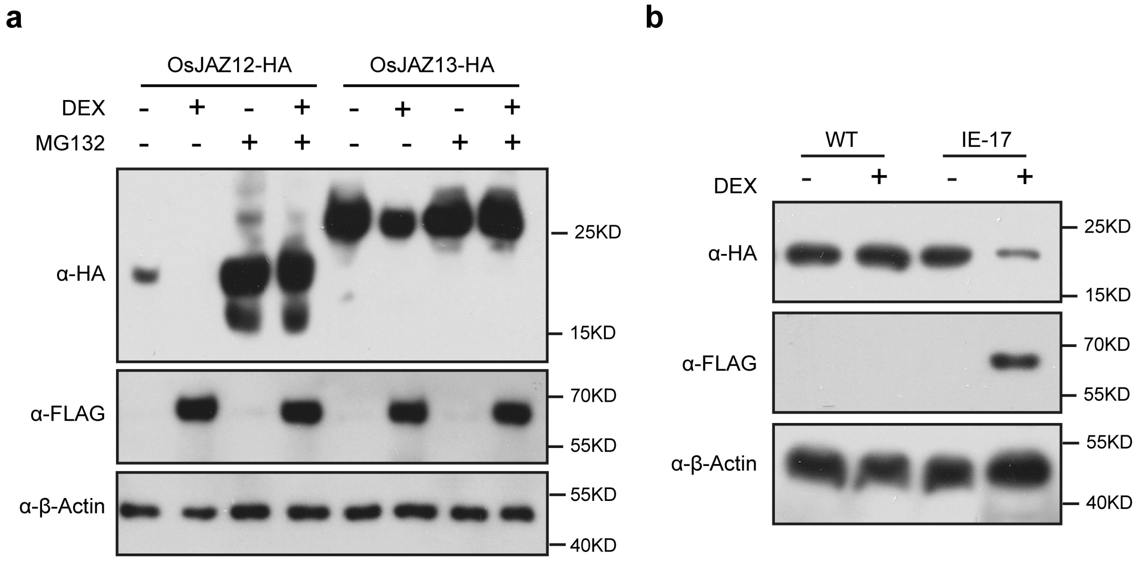

Supplementary Figure 6. XopC2 promotes OsJAZ protein degradation in rice protoplasts. a, The accumulation of OsJAZ12/13-HA was greatly reduced in XopC2-FLAG-expressing transgenic rice protoplasts. Individual OsJAZ-HA proteins were transiently expressed in rice protoplasts isolated from IE-17 transgenic seedlings under mock and DEX treatments for 12 hours. Immunoblotting analyses were performed to detect OsJAZs-HA, XopC2-FLAG and *β*-OsActin (as a protein loading control). MG132, a proteasome inhibitor, was added to inhibit 26S proteasome-mediated protein degradation. **b,** Reduced OsJAZ9-HA accumulation in rice protoplasts was caused by XopC2-FLAG expression, not by DEX treatment. Rice protoplasts prepared from the wild-type (WT) and IE-17 transgenic seedlings were transfected with pUC19-*35S::OsJAZ9-3HA* and were treated with DEX or mock solution for 12 hours. OsJAZ9-HA, XopC2-FLAG and *β*-OsActin were detected by immunoblotting. *β*-OsActin indicates total protein loading. The experiments were independently repeated for 3 times with similar results in **a** and **b**.

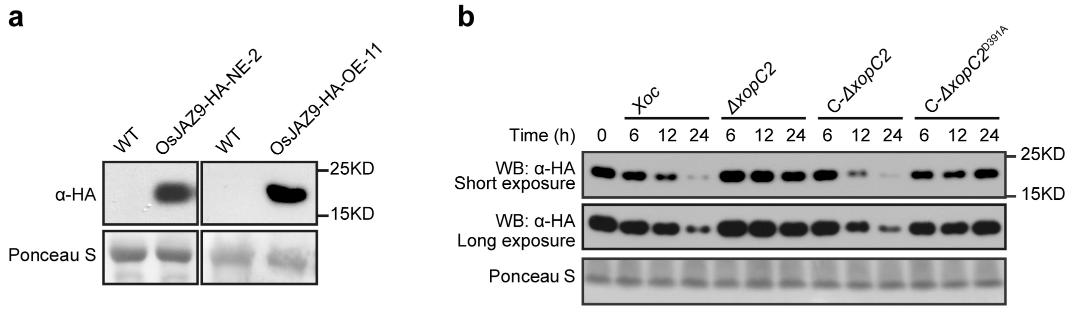

Supplementary Figure 7. XopC2-induced degradation of OsJAZ9 in the OsJAZ9-HA-expressing transgenic rice plants. a, Expression of OsJAZ9-HA driven by the native and 35S promoters in the OsJAZ9-HA-NE-2 and OsJAZ9-HA-OE-11 transgenic rice lines, respectively, were detected by immunoblotting with an anti-HA antibody (α-HA). Lower panel, the same blots were stained with Ponceau S to show protein loading. WT, the wild-type Nipponbare plant. **b**, OsJAZ9-HA was rapidly degraded during *Xoc* infection, but remained relatively stable after *ΔxopC2* infection. Three-week-old transgenic seedlings constitutively expressing OsJAZ9-HA were sprayed with the strains RS105, *ΔxopC2*, C-*ΔxopC2* complemented with a wild-type copy of *xopC2* or C-*ΔxopC2*^{D391A} complemented with a kinase-defective copy of *xopC2*. OsJAZ9-HA was detected by immunoblotting at the indicated time-points post inoculation. The experiments were independently repeated for 3 times with similar results in **a** and **b**.

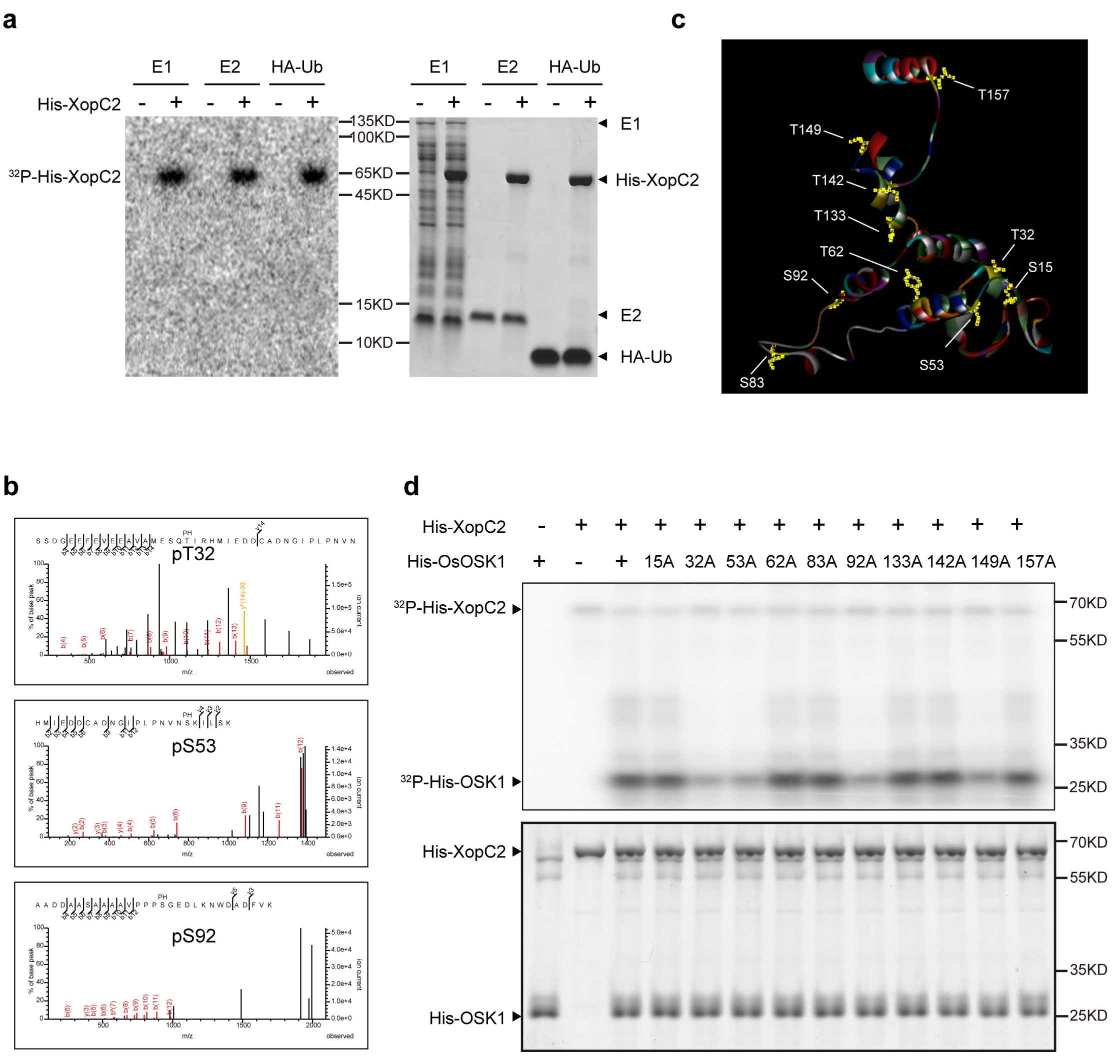

Supplementary Figure 8. The mutations at Thr^{32}, Ser^{53}, Ser^{92} and Thr^{149} residues attenuate XopC2-catalyzed OSK1 phosphorylation. a, *In vitro* kinase assays showed that His6-SUMO-OsUBA1 (E1), UBCH5α (E2) or HA-ubiquitin were not phosphorylated by XopC2. Left panel, protein phosphorylation was detected by autoradiography. Right panel, protein loading was indicated by CBB staining. **b,** OSK1 was phosphorylated at the Thr^{32}, Ser^{53} and Ser^{92} residues as revealed by LC-MS/MS analysis. After *in vitro* phosphorylation reaction, His6-XopC2 and His6-OSK1 were digested with trypsin, and were then subject to LC-MS/MS analysis. Thr^{32}, Ser^{53} and Ser^{92} phosphorylation was identified by MS/MS spectra of three OSK1 peptides including

SSDGEEFEVEEAVAMESQ(phosphorylated)TIRHMIEDDCADNGIPLPNVN (upper panel), HMIEDDCADNGIPLPNVN(phosphorylated)SKILSK (middle panel) and AADDAASAAAAVPPP(phosphorylated)SGEDLKNWDADFVK (lower panel). **c,** Structure modeling of OSK1. The 3-D structure of OSK1 was predicted via homology modeling with SWISS-MODEL. The image was generated with Accelrys Discovery Studio 2.5. The labeled Ser and Thr residues exposed on the surface of 3-D structure are predicted to be phosphosites. **d,** XopC2-mediated phosphorylation of $OSK1^{T32A}$, $OSK1^{S53A}$, $OSK1^{S92A}$ and $OSK1^{T149A}$ was greatly attenuated compared with OSK1 phosphorylation. In *in vitro* phosphorylation assays, His6-XopC2 was incubated with OSK1 and alanine-replaced mutants at candidate phosphosites (Ser^{15}, Thr^{32}, Ser^{53}, Tyr^{62}, Ser^{83}, Ser^{92}, Thr^{133}, Thr^{142}, Thr^{149} and Thr^{157}) individually. Upper panel, protein phosphorylation was detected by autoradiography; lower panel, protein loading was indicated by CBB staining. The experiments were independently repeated for 3 times with similar results in **a** and **d**.

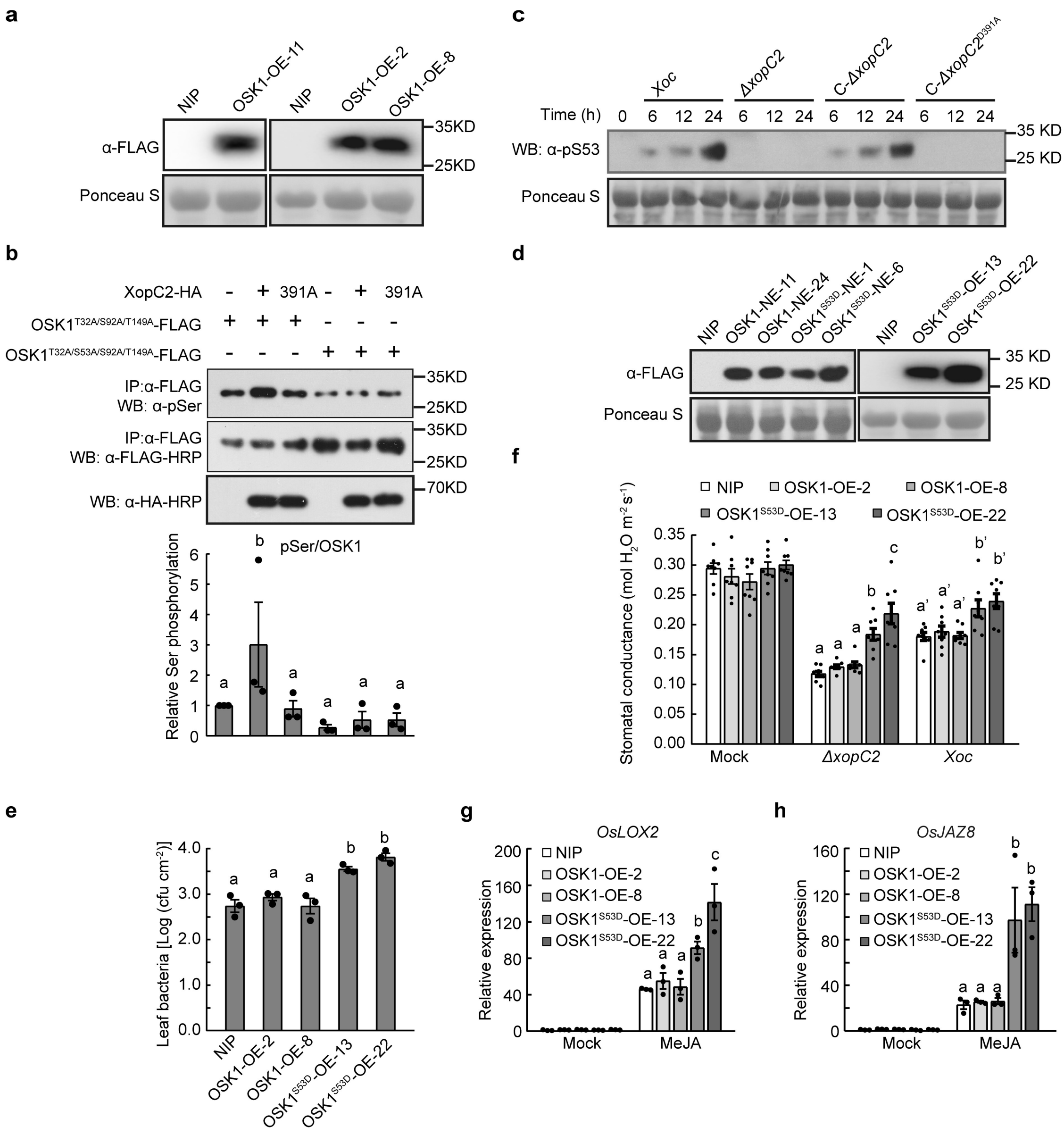

Supplementary Figure 9. XopC2 phosphorylates OSK1 at Ser^{53} and enhances JA signaling. a, Expression of OSK1-FLAG driven by a maize ubiquitin promoter in the transgenic rice lines was detected using an anti-FLAG antibody (α-FLAG). Protein loading is indicated by Ponceau S staining. NIP, Nipponbare. **b,** XopC2 preferentially phosphorylated OSK1 at Ser^{53} when co-expressed in rice protoplasts. OSK1$^{T32A/S92A/T149A}$-FLAG and OSK1$^{T32A/S53A/S92A/T149A}$-FLAG were expressed alone, with XopC2-HA or XopC2^{D391A}-HA in rice protoplasts. OSK1-FLAG variants were immunoprecipitated from protein extracts of transfected protoplasts with

anti-FLAG M2 affinity beads. Phosphorylated and total OSK1-FLAG was quantified after immunoblotting as described in Fig. 4**b**. Data are presented as means ± SE (n = 3 independent experiments). The letters (a, b) indicate a statistically significant difference in signal intensity (one-way ANOVA, Tukey's honest significance test). **c**, Time course assay on OSK1 phosphorylation at Ser^{53} after infection of different *Xoc* strains. The OsJAZ9-HA-NE-2 transgenic seedlings were inoculated as described in Fig. 3**d**. OSK1 phosphorylation at Ser53 was detected by immunoblotting with an anti-pSer53 polyclonal antibody. **d**, Expression of $OSK1^{S53D}$-FLAG driven by native promoter (left) and a maize ubiquitin promoter (right) in the transgenic rice lines was detected by western blotting with an anti-FLAG antibody (α-FLAG). Protein loading is indicated by Ponceau S staining. NIP, Nipponbare. **e**, Bacterial population sizes in the *ΔxopC2*-inoculated wild-type and *OSK1* transgenic seedlings. The transgenic rice lines OSK1-OE-2/8 and $OSK1^{S53D}$-OE-13/22 were spray-inoculated as described in Fig. 2a. Data are shown as means ± SE (n = 3 technical replicates). The letters (a, b) indicate a statistically significant difference in bacterial population sizes (one-way ANOVA, Tukey's honest significance test). **f**, Stomatal conductance of the wild-type and *OSK1* transgenic plant leaves after *ΔxopC2* inoculation. Data are shown as means ± SE (n = 8 technical replicates). The letters (a, b) indicate a statistically significant difference in Gs of the wild-type and different transgenic lines (one-way ANOVA, Tukey's honest significance test). **g-h,** MeJA-induced expression of the JA-responsive genes *OsLOX2* (**g**) and *OsJAZ8* (**h**) in the wild-type, *OSK1-* and *$OSK1^{S53D}$*-overexpressing transgenic plants. Data are shown as means ± SE (n = 3 technical replicates). The letters (a, b) indicate a statistically significant difference in relative gene expression levels (one-way ANOVA, Duncan's multiple range test). These experiments were all independently repeated for 3 times with similar results.

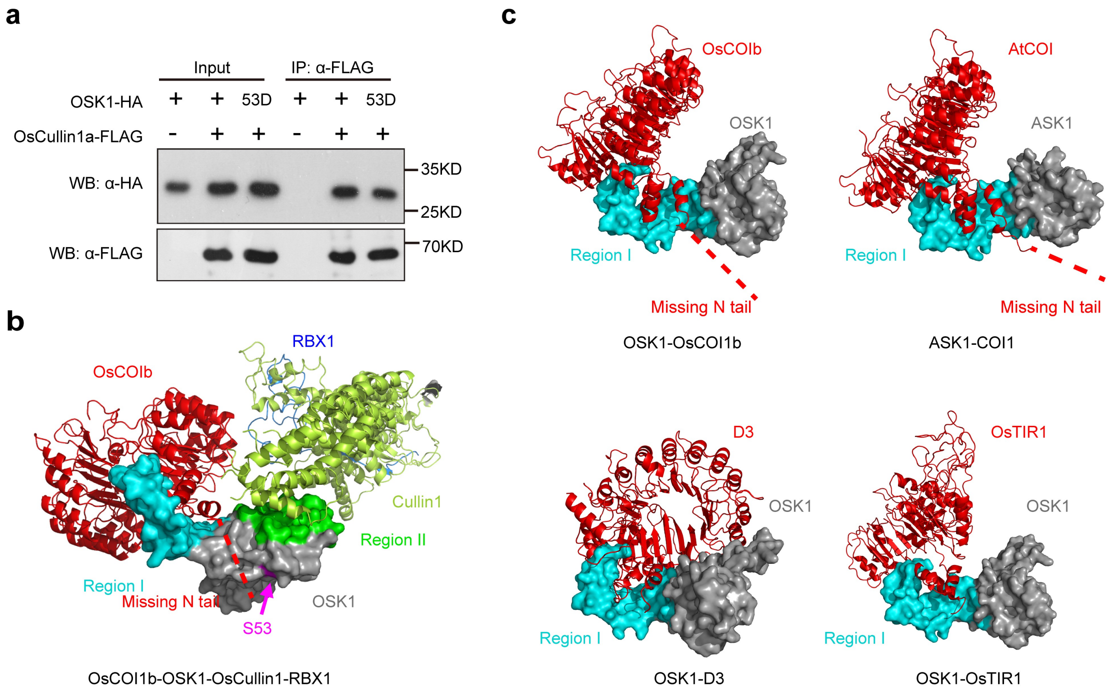

Supplementary Figure 10. The S53D mutation enhances OSK1 binding to OsCOI1b but not to OsCullin1a. a, Co-IP assays to detect the interactions of OsCullin1a-FLAG with OSK1-HA and with OSK1^{S53D}-HA in rice protoplasts. OsCullin1a-FLAG was transiently expressed alone or together with OSK1-HA and OSK1^{S53D}-HA individually in rice protoplasts. The input proteins and immunocomplex were detected by immunoblotting using anti-HA and anti-FLAG antibodies. IP, immunoprecipitation; α-HA, anti-HA antibody; α-FLAG, anti-FLAG antibody. The experiments were independently repeated for 3 times with similar results. **b,** The 3-D structure of OsCOI1b-OSK1-Cullin1-RBX1 constructed via homology modeling with the ASK1-COI1-JAZ (PDB: 3OGM) and Cul1-Rbx1-Skp1-F box^{Skp2} (PDB: 1LDK) complexes as templates. The conserved regions I (cyan) and II (green) of OSK1 interact with OsCOI1b and Cullin1, respectively. The Ser^{53} residue was colored with magenta. The N-terminal flexible tail of OsCOI1b missing from the structure was arbitrarily labelled with a dotted red line. **c,** The 3-D structures of OSK1-OsCOI1b, OSK1-D3 and OSK1-OsTIR1 complexes constructed by

homology modelling using ASK1-COI1-JAZ (PDB: 3OGM), AtD14-D3-ASK1 (PDB: 5HZG) and TIR1-ASK1(PDB: 2P1M) crystal structures as templates, respectively. OsCOI1b and COI1 contain a special extended N-terminal tail that was missing from the predicted structure and was arbitrarily drawn, while D3 and OsTIR1 do not. The structures were generated with PyMOL.

Supplementary Table 1, Primers, bacteria strains, plants and reagent in this study.

PRIMERS	SEQUENCE(5'-3')
xopC2-pET28a-*Nde*I-F	AAACATATGGAATCTCGTATCACCCC
xopC2-pET28a/pGEX4T-3-*Xho*I-R	AAACTCGAGTCATTTCCGGGCTTTCTCAACCA
xopC2-pGEX-4T-3-F	AAAGAATTCCATGGAATCTCGTATCACCCC
xopC2-D391A-F	GACCCTGGGCGAAGCGTCTGCTCTAGGCCCTGACAATATGC
xopC2-D391A-R	GCATATTGTCAGGGCCTAGAGCAGACGCTTCGCCCAGGGTC
xopC2-N396A-F	CGTCTGATCTAGGCCCTGACGCTATGCTGGTCATTCCCGGAG
xopC2-N396A-R	CTCCGGGAATGACCAGCATAGCGTCAGGGCCTAGATCAGACG
OsJAZ9-pET32b-*Kpn*I-F	AAAGGTACCATGGCGTCGACGGATCCCAT
OsJAZ9-pET32b-*Eco*RI-R:	AAAGAATTCTCAGCGCGAGTGCATGTGTC
OsJAZ9-FLAG-pET32b-*Eco*RI-R	AAAGAATTCTCACTTATCGTCGTCATCCTTGTAATCGCGCGAGTGCATGTGTC CAA
OsUBA3-pET28a-*Bam*HI-F:	AAAGGATCCATGTCCTCCCCCGACGAGG
OsUBA3-pET28a-*Hin*dIII-R:	AAAAAGCTTCAAGAACTCTCATCCATTTTC
OsAXR1-pET28a-*Bam*HI-F:	AAAGGATCCATGGCCGCCGCCAC
OsAXR1-pET28a-*Hin*dIII-R:	AAAAAGCTTATAACGCCAAAACTTGAG
OsUBC12-pET28a-*Bam*HI-F	AAAGGATCCATGCTTAATCTTATCAAAAT
OsUBC12-pET28a-*Xho*I-R	AAACTCGAGTCATGCACATCTTGGGAAAT
OsDCN1-pET28a-*Nde*I-F	AAACATATGATGCAATTTTGGCATAAGC
OsDCN1-pET28a-*Xho*I-R	AAACTCGAGTCACTTCCTAAGCTGAACGA
OsRBX1-pET28a-*Nde*I-F	AAACATATGATGGACAAGGGCGACGTCGC
OsRBX1-pET28a-*Xho*I-R	AAACTCGAGCTAGTGACCATACTTCTGGA
OsRUB1Δ77-pET28a-*Nde*I-F	AAACATATGATCAAGGTTAAGACCCT
OsRUB1-pET28a-*Xho*I-R	AAACTCGAGCTAACCACCCCTCAGAGCAAG
Upl1-4T-3-BamHI-F	AAAGGATCCCTTGTTCCTGAATTAAATGA
Upl1-4T-3-XhoI-R	AAACTCGAGCTATTTTAAAGCGTCGGTTA
OsCullin1a-pColdTF-*Nde*I-F	AAACATATGATGGCGGGGCAGGAGCGGAG
OsCullin1a-pColdTF-*Eco*RI-R	AAAGAATTCTCAAGCCAGGTATCTGTACA
TF-SUMO-HR-F	GTGGTGGTATCGAAGGTAGGCATATGAATTGGAGCCACCCGCA
TF-SUMO-OsCullin1a-HR-R	GCAGAGATTACCTATCTAGACTGCAGTCAAGCCAGGTATCTGTACA

SUMO-OsCullin1a-mid-F	ACCGCGAACAGATTGGAGGCATGGCGGGGCAGGAGCGGAG
SUMO-OsCullin1a-mid-R	ATTACCTATCTAGACTGCAGTCAAGCCAGGTATCTGTACA
OsCO1b-pColdTF-*Bam*HI-F	AAAGGATCCATGGGAGGGGAGGCACCGGAG
OsCO1b-pColdTF-*Sal*I-R	AAAGTCGACTCACGCAGGATACAAAGGAA
OsCoi1b-R9A-F:	GGAGGCACCGGAGGCGGCGCGGTTGGACCGCGCGATG
OsCoi1b-R9A-R:	CATCGCGCGGTCCAACCGCGCCGCCTCCGGTGCCTCC
OsCoi1b-R10A-F:	GGAGGCACCGGAGGCGCGGGCGTTGGACCGCGCGATG
OsCoi1b-R10A-R:	CATCGCGCGGTCCAACGCCCGCGCCTCCGGTGCCTCC
OsCoi1b-R13A-F:	GGCGCGGCGGTTGGACGCCGCGATGAGCTTCGGC
OsCoi1b-R13A-R:	GCCGAAGCTCATCGCGGCGTCCAACCGCCGCGCC
TF-SUMO-OsCoi1b-HR-R	ATTACCTATCTAGACTGCAGGTCGACTCACGCAGGATACAAAGGAA
SUMO-OsCO1b-mid-F	ACCGCGAACAGATTGGAGGCATGGGAGGGGAGGCACCGGA
SUMO-OsCO1b-mid-R	TCCGGTGCCTCCCCTCCCATGCCTCCAATCTGTTCGCGGT
OsUBA1-pCold-SUMO-HR-F:	GCTCACCGCGAACAGATTGGAGGCATGAGGTGCTTACGGTTTCT
OsUBA1-pCold-SUMO-HR-R:	CTATCTAGACTGCAGGTCGACCTATCGGAAGTAAACTGAGA
OSK1-pET28a-*Nde*I-F	AAACATATGATGGCGGCTGAGGGAGAGAA
OSK1-pET28a-*Eco*RI-R	AAAGAATTCCTACTCAAAAGCCCACTGGT
OSK1-4T-3-*Bam*HI-F	AAAGGATCCATGGCGGCTGAGGGAGAGAA
OSK1-4T-3-*Xho*I-R	AAACTCGAGCTACTCAAAAGCCCACTGGT
OSK1-S15A-F	ATCACCCTGAAGAGCGCCGACGGGGAGGAGTT
OSK1-S15A-R	AACTCCTCCCCGTCGGCGCTCTTCAGGGTGAT
OSK1-T32A-F	GGCGATGGAGTCGCAGGCCATCCGCCACATGAT
OSK1-T32A-R	ATCATGTGGCGGATGGCCTGCGACTCCATCGCC
OSK1-S53A-F	GCTCCCCAACGTCAACGCCAAGATCCTCTCCAA
OSK1-S53A-R	TTGGAGAGGATCTTGGCGTTGACGTTGGGGAGC
OSK1-Y62A-F	CTCCAAGGTCATCGAGGCCTGCAACAAGCACGTGC
OSK1-Y62A-R	GCACGTGCTTGTTGCAGGCCTCGATGACCTTGGAG
OSK1-S83A-F	GCCGCCGACGACGCCGCGGCCGCCGCCGCAGCCGTGCC
OSK1-S83A-R	GGCACGGCTGCGGCGGCGGCCGCGGCGTCGTCGGCGGC
OSK1-S92A-F	AGCCGTGCCGCCGCCCGCCGGCGAGGACCTCAAG
OSK1-S92A-R	CTTGAGGTCCTCGCCGGCGGGCGGCGGCACGGCT
OSK1-T133A-F	GGACCTTACTTGCCAGGCTGTTGCTGACATGATC
OSK1-T133A-R	GATCATGTCAGCAACAGCCTGGCAAGTAAGGTCC
OSK1-T142A-F	CATGATCAAGGGGAAGGCTCCTGAGGAGATCCGC
OSK1-T142A-R	GCGGATCTCCTCAGGAGCCTTCCCCTTGATCATG
OSK1-T149A-F	TGAGGAGATCCGCAAGGCCTTCAACATCAAGAAC
OSK1-T149A-R	GTTCTTGATGTTGAAGGCCTTGCGGATCTCCTCA
OSK1-T157A-F	CATCAAGAACGACTTCGCCCCTGAGGAGGAAGAG
OSK1-T157A-R	CTCTTCCTCCTCAGGGGCGAAGTCGTTCTTGATG
OSK1-T32D-F	GGCGATGGAGTCGCAGGACATCCGCCACATGAT
OSK1-T32D-R	ATCATGTGGCGGATGTCCTGCGACTCCATCGCC
OSK1-S53D-F	GCTCCCCAACGTCAACGACAAGATCCTCTCCAA
OSK1-S53D-R	TTGGAGAGGATCTTGTCGTTGACGTTGGGGAGC

OSK1-S92D-F	AGCCGTGCCGCCGCCCGACGGCGAGGACCTCAAG
OSK1-S92D-R	CTTGAGGTCCTCGCCGTCGGGCGGCGGCACGGCT
OSK1-T149D-F	TGAGGAGATCCGCAAGGACTTCAACATCAAGAAC
OSK1-T149D-R	GTTCTTGATGTTGAAGTCCTTGCGGATCTCCTCA
xopC2-3FLAG-pUC19-35S-*Xho*I-F:	AACTCGAGATGGAATCTCGTATCACCCC
xopC2-3FLAG-pUC19-35S-*Csp*45 I-R:	ATATTCGAATTTCCGGGCTTTCTCAACCA
*xopC2-Kpn*I-F	AAAGGTACCATGGAATCTCGTATCACC
*XopC2-Xba*I-R	AAATCTAGATTTCCGGGCTTTCTCAACC
OSK1-3HA-pUC19-35S-KpnI-F	AAAGGTACCATGGCGGCTGAGGGAGAGAA
OSK1-3HA-pUC19-35S-XbaI-R	AAATCTAGACTCAAAAGCCCACTGGTTCT
*OSK1-FLAG-Kpn*I-F	AAAGGTACCATGGCGGCTGAGGGAGAGAA
*OSK1-FLAG-Xba*I-R	AAATCTAGACTACTTATCGTCGTCATCCTTGTAATCCTCAAAAGCCCACTGGTTCT
OsCOI1b-3FLAG-pUC19-35S-*Kpn*I-F :	AAAGGTACCATGGGAGGGGAGGCACCGGA
OsCOI1b-3FLAG-pUC19-35S-*Sal*I -R:	AAAGTCGACCGCAGGATACAAAGGAACCA
OsCullin1a-pHBT-*Bam*HI-F	AAAGGATCCATGGCGGGGCAGGAGCGGAG
OsCullin1a-pHBT-*Stu*I-R	AAAAGGCCTAGCCAGGTATCTGTACACAT
OsCullin1a-FLAG-pHBT-*Stu*I-R	AAAAGGCCTTCACTTATCGTCGTCATCCTTGTAATCAGCCAGGTATCTGTACACAT
OsRBX1-pHBT-*Bam*HI-F	AAAGGATCCATGGACAAGGGCGACGTCGC
OsRBX1-pHBT-*Stu*I-R	AAAAGGCCTGTGACCATACTTCTGGAACT
OsJAZ7--3HA-pUC19-35S-*Kpn*I-F:	AAAGGTACCATGGCGGCTTCCGCGAGGCC
OsJAZ7-3HA-pUC19-35S-*Xba*I-R:	AAATCTAGAAGAAAGGGCAGAGTAATTAC
OsJAZ9-3HA-pUC19-35S-*Kpn*I-F	AAAGGTACCATGGCGTCGACGGATCCCAT
OsJAZ9-3HA-pUC19-35S-*Xba*I-R	AAATCTAGAGCGCGAGTGCATGTGTCCAA
OsJAZ9-NP-*Eco*RI-F	AAAGAATTCGTGAAGATTGGTTGAAGATGG
OsJAZ9-NP-*Kpn*I-R	AAAGGTACCTCAAGCGTAGTCTGGGACGTCGTATGGGTAGCGCGAGTGCATGTGTCC
OsJAZ12-3HA-pUC19-35S-*Kpn*I-F	AAAGGTACCATGCGGGAGCGCCAGCAGCC
OsJAZ12-3HA-pUC19-35S-*Xba*I-R	AAATCTAGAGAGCCCGAGCCATGTCGCCG
OsJAZ13-3HA-pUC19-35S-*Kpn*I-F	AAAGGTACCATGGCGGCGGAGGCGGCGGC
OsJAZ13-3HA-pUC19-35S-*Xba*I-R	AAATCTAGAGAGCGCGAGCGCGAGGTGGT
xopC2-pENTR-F	CACCATGAAGCGCGAGTACCAAGAAGCCG
xopC2-pENTR-R	CGCCGCGGCGACGCGCCATG
OSK1-PC1305-*Kpn*I-F	AAAGGTACCATGGCGGCTGAGGGAGAGAA
OSK1-PC1305-*Hind*III-R	CCCAAGCTTCTCAAAAGCCCACTGGTTCT
OSK1-NP-F	TAGCGCTACCGGTCGCCACCGAGCTCTCTTTGGGAGAGGACCTACTC
OSK1-NP-R	TTCTCTCCCTCAGCCGCCATCGATCGAGCCCTCCGAAATC
xopC2-HA-pVSP61-*Eco*RI-F	AAAGAATTCTTCCAGCCGCCGATACCAAG
xopC2-HA-pVSP61-mid-R	GGGGTGATACGAGATTCCAT

xopC2-HA-pVSP61-mid-F	ATGGAATCTCGTATCACCCC
xopC2-HA-pVSP61-*Sal*I-R	AAAGTCGACTCACGCATAGTCAGGAACATCGTATGGGTATTTCCGGGCTTTCTCAACCA
nLuc-OSK1-HR-F	GGAGAGAACACGGGGGACGAGCTCATGGCGGCTGAGGGAGAGAA
nLuc-OSK1-HR-R	CGGGACGCGTACGAGATCTGGTCGACCTCAAAAGCCCACTGGTTCT
cLUC-xopC2-HR-F	CTCGTACGCGTCCCGGGGCGGTACCATGGAATCTCGTATCACC
cLUC-xopC2-HR-R	CGATGATACGAACGAAAGCTCTGCAGTCATTTCCGGGCTTTCTCA
OsActin1-qRT-F	TCCATCTTGGCATCTCTCAG
OsActin1-qRT-R	GTACCCGCATCAGGCATCTG
OsLOX2-qRT-F	CTGCCGTACCAGCTGATGAAGC
OsLOX2-qRT-R	AGATTTGGGAGTGACATATTGGTT
OsJAZ8-qRT-F	GAAAGTGCAAGTGAGGCAGC
OsJAZ8-qRT-R	ATCCTTGACCTTGGTGGACG
D10-qRT-F	CGTGGCGATATCGATGGT
D10-qRT-R	CGACCTCCTCGAACGTCTT
OsIAA9-qRT-F	AAGAAAATGGCCAATGATGATCA
OsIAA9-qRT-R	CCCATCACCATCCTCGTAGGT
OsICS1-qRT1-F	GTACACGAGCAAGGGGGAAA
OsICS1-qRT1-R	TGGACAACCACATCATCGCA
OsPAL1-qRT-F	ACATCTACGGCGTCACCAC
OsPAL-qRT-R	GAAGATTCCGGCGTTGAG
**BACTERIA STRAINS**	**FEATURES**
Escherichia coli DH 5α	F-，φ80dlacZΔM15，Δ(lacZYA-argF)U169，deoR，recA1，endA1，hsdR17(rk-，mk)，phoA，supE44，λ-，thi-1，gyrA96，relA1
Escherichia coli BL21 (DE3)	F-, ompT, hsdS (rBB-mB-), gal, dcm (DE3)
***Xanthomonas oryzae* pv. *oryzicola* Strains**	
RS105	X. oryzae pv.*oryzicola*，Rif^r
ΔxopC2	In frame deletion of *xopC2* gene
C-*ΔxopC2*	Complementation，*xopC2*-HA cloned in pVSP61，Kar
C-*ΔxopC2*^{D391A}	Complementation，*xopC2*-HA cloned in pVSP61，Kar
Agrobacterium tamaficium EHA105	C58 (rif R) Ti pEHA105 (pTiBo542DT-DNA) (strepR) Succinamopine
**PLANTS**	**FEATURES**
Oryza sativa L. ssp. *japonica* cv. *Nipponbare*	Wild-type rice plant
XopC2-OE-1,10	Transgenic rice plants carrying 35S promotor-driven *xopC2-3FLAG* in Nipponbare background
XopC2-IE-17,37	Transgenic rice lines carrying DEX inducible promotor-driven *xopC2-3FLAG* in Nipponbare background
XopC2-IE-D391A-2	Transgenic rice lines carrying DEX inducible promotor-driven *xopC2*^{D391A}-3FLAG in Nipponbare background
XopC2-IE-N396A-14	Transgenic rice lines carrying DEX inducible promotor-driven *xopC2*^{N396A}-3FLAG in Nipponbare background

OsJAZ9-HA-OE-11	Transgenic rice lines carrying 35S promotor-driven *OsJAZ9-3HA* in Nipponbare background
OsJAZ9-HA-NE-2	Transgenic rice lines carrying Native promotor-driven *OsJAZ9-3HA* in Nipponbare background
OSK1-FLAG-OE-2, 8, 11	Transgenic rice lines carrying Ubi promotor-driven *OSK1-3FLAG* in Nipponbare background
OSK1^{S53D}-FLAG-OE-13,22	Transgenic rice lines carrying Ubi promotor-driven *OSK1^{S53D}-3FLAG* in Nipponbare background
OSK1-FLAG-NE-11, 24	Transgenic rice lines carrying native promotor-driven *OSK1-3FLAG* in Nipponbare background
OSK1^{S53D}-FLAG-NE-1,6	Transgenic rice lines carrying native promotor-driven *OSK1^{S53D}-3FLAG* in Nipponbare background
**ANTIBODIES**	**SOURCE**
anti-FLAG M2 monoclonal antibody	Sigma-Aldrich, F1804
HRP-conjugated anti-FLAG M2 monoclonal antibody	Sigma-Aldrich, A8592
anti-FLAG M2 affinity gel	Sigma-Aldrich, A2220
HRP-conjugated anti-HA monoclonal antibody	Roche,11667475001
HRP-conjugated anti-His monoclonal antibody	CWBIO, CW0285
anti-GST monoclonal antibody	CWBIO, CW0084
Anti-β-Actin monoclonal antibody	CWBIO, CW0264
anti-phosphoserine polyclonal antibody	Millipore, AB1603
anti-OSK1^{pS53} polyclonal antibody	Generated by immunizing rabbits with a phosphopeptide, Ac-C-LPNVN(pS)KILSK-NH_2 (Abmart, Shanghai, China)
**CHEMICALS AND RECOMBINANT PROTEINS**	**SOURCE**
ATP	Sigma-Aldrich, A6559
γ-^{32}P-ATP	PerkinElmer, NEG502A
MG132	Millipore, 474790
Coronatine	Sigma-Aldrich, C8115
Protease inhibitor cocktail	CWBIO, CW2200
MeJA	Sigma-Aldrich, 392707
rac-GR24	Coolaber, CR9420
NAA	Sigma-Aldrich, N0640
UBA1	Boston Biochem, E-305-025
UBCH5α	Boston Biochem, E2-616-100
HA-Ub	Boston Biochem, U-110-01M
