# Supplementary material for: A bacterial kinase phosphorylates OSK1 to suppress stomatal immunity in rice
Source: Nat Commun. 2021 Sep 16;12:5479. doi: 10.1038/s41467-021-25748-4 (PMC8445998; doi:10.1038/s41467-021-25748-4)
Supplement: Supplementary file 3 — Reporting summary [file 41467_2021_25748_MOESM3_ESM.pdf]

nature portfolio

Corresponding author(s): Wenxian Sun

Last updated by author(s): Aug 21, 2021

Reporting Summary

Nature Portfolio wishes to improve the reproducibility of the work that we publish. This form provides structure for consistency and transparency in reporting. For further information on Nature Portfolio policies, see our Editorial Policies and the Editorial Policy Checklist.

Statistics

For all statistical analyses, confirm that the following items are present in the figure legend, table legend, main text, or Methods section.

n/a	Confirmed	
☐	☒	The exact sample size (*n*) for each experimental group/condition, given as a discrete number and unit of measurement
☐	☒	A statement on whether measurements were taken from distinct samples or whether the same sample was measured repeatedly
☐	☒	The statistical test(s) used AND whether they are one- or two-sided<br>*Only common tests should be described solely by name; describe more complex techniques in the Methods section.*
☐	☒	A description of all covariates tested
☐	☒	A description of any assumptions or corrections, such as tests of normality and adjustment for multiple comparisons
☐	☒	A full description of the statistical parameters including central tendency (e.g. means) or other basic estimates (e.g. regression coefficient) AND variation (e.g. standard deviation) or associated estimates of uncertainty (e.g. confidence intervals)
☐	☒	For null hypothesis testing, the test statistic (e.g. *F*, *t*, *r*) with confidence intervals, effect sizes, degrees of freedom and *P* value noted<br>*Give P values as exact values whenever suitable.*
☒	☐	For Bayesian analysis, information on the choice of priors and Markov chain Monte Carlo settings
☐	☒	For hierarchical and complex designs, identification of the appropriate level for tests and full reporting of outcomes
☒	☐	Estimates of effect sizes (e.g. Cohen's *d*, Pearson's *r*), indicating how they were calculated

Our web collection on statistics for biologists contains articles on many of the points above.

Software and code

Policy information about availability of computer code

Data collection	Phosphorylated sites were analyzed by MS/MS spectra data with Mascot Distiller (Matrix Science; version 2.4); Stomatal conductance was measured using a photosynthesis system (Model LI-6400XT, Li-Cor Inc., Lincoln, NE, USA); The chlorophyll content was measured by a spectrophotometer(Thermo Scientific, Genesys 180 UV-visible spectrophotometer); Microscale thermophoresis assay was performed using a NanoTemper MonolithTM NT.115 instrument (Munich, Germany), and the data was analyzed with MO. Affinity analysis software. Quantitative RT-PCR data was collected with an ABI PRISM® 7500 Sequence Detection System (Applied Biosystems, Carlsbad, CA, USA).
Data analysis	Data was analyzed with Graphpad Prism 8.0. Statistical analysis was performed using SPSS software system (vision 19.0, SPSS, Inc., Chicago, IL).

For manuscripts utilizing custom algorithms or software that are central to the research but not yet described in published literature, software must be made available to editors and reviewers. We strongly encourage code deposition in a community repository (e.g. GitHub). See the Nature Portfolio guidelines for submitting code & software for further information.

Data

Policy information about availability of data

All manuscripts must include a data availability statement. This statement should provide the following information, where applicable:

- Accession codes, unique identifiers, or web links for publicly available datasets
- A description of any restrictions on data availability
- For clinical datasets or third party data, please ensure that the statement adheres to our policy

All data are available from the corresponding author and all unique materials generated are available from the authors. Source data for Figures and Supplementary Figures are provided in a Source data file.

Field-specific reporting

Please select the one below that is the best fit for your research. If you are not sure, read the appropriate sections before making your selection.

☒ Life sciences ☐ Behavioural & social sciences ☐ Ecological, evolutionary & environmental sciences

For a reference copy of the document with all sections, see nature.com/documents/nr-reporting-summary-flat.pdf

Life sciences study design

All studies must disclose on these points even when the disclosure is negative.

Sample size	Before each experiment, we performed pre-experiments to determinate the minimal sample size (n) by calculating with the forma: n = (Z2/α2 σ2) / E2. Z, Confidence interval; α=0.05; σ, Standard Deviation; E, expected variance, was set as 0.05.
Data exclusions	Strictly following the data analysis guidelines of manufacturer's manual, we excluded no more than 2 abnormal data in MST assay. In all other experiments, we used intact data to perform assays, and did not exclude any data.
Replication	In the assay of virulence, stomatal conductance, chlorophyll content, hormone content, and agronomic traits, we were blinded to group allocation and data collection.
Randomization	In each experiment, all plant materials were grown in the same conditions, and for each plant lines, seedlings were randomly grouped to performed assays.
Blinding	In the assay of virulence, stomatal conductance, chlorophyll content, hormone content, and agronomic traits, we were blinded to group allocation and data collection.

Reporting for specific materials, systems and methods

We require information from authors about some types of materials, experimental systems and methods used in many studies. Here, indicate whether each material, system or method listed is relevant to your study. If you are not sure if a list item applies to your research, read the appropriate section before selecting a response.

Materials & experimental systems

n/a	Involved in the study	
☐	☒	Antibodies
☒	☐	Eukaryotic cell lines
☒	☐	Palaeontology and archaeology
☒	☐	Animals and other organisms
☒	☐	Human research participants
☒	☐	Clinical data
☒	☐	Dual use research of concern

Methods

n/a	Involved in the study	
☒	☐	ChIP-seq
☒	☐	Flow cytometry
☒	☐	MRI-based neuroimaging

Antibodies

Antibodies used	Anti-FLAG M2 monoclonal antibody (Sigma-Aldrich, F1804); HRP-conjugated anti-FLAG M2 monoclonal antibody (Sigma-Aldrich, A8592); HRP-conjugated anti-HA monoclonal antibody (Roche,11667475001); HRP-conjugated anti-His monoclonal antibody (CWBIO, CW0285); anti-GST monoclonal antibody (CWBIO, CW0084); anti-beta-Actin monoclonal antibody (CWBIO, CW0264); Anti-phosphoserine polyclonal antibody (Millipore, AB1603); Anti-OSK1pS53 polyclonal antibody generated by immunizing rabbits with a phosphopeptide, Ac-C-LPNVN(pS)KILSK-NH2 (Abmart, Shanghai, China).
Validation	Anti-OSK1pS53 polyclonal antibody was generated by immunizing rabbits with a phosphopeptide, Ac-C-LPNVN(pS)KILSK-NH2 (Abmart, Shanghai, China), and this antibody was used to detected phosphorylation of Ser53 amino acid residue in rice OSK1 protein. Other antibodies were all produced by commercial manufacturers, and the validations of each antibody was described in the relevant manufacturer webpages as fellow:<br>Anti-FLAG M2 monoclonal antibody (Sigma-Aldrich, F1804), https://www.sigmaaldrich.cn/CN/en/product/sigma/f1804?context=product<br>HRP-conjugated anti-FLAG M2 monoclonal antibody (Sigma-Aldrich, A8592), https://www.sigmaaldrich.cn/CN/en/product/sigma/a8592?context=product<br>HRP-conjugated anti-HA monoclonal antibody (Roche,11667475001), https://www.sigmaaldrich.cn/CN/en/product/roche/11667475001?context=product<br>HRP-conjugated anti-His monoclonal antibody (CWBIO, CW0285), https://www.cwbio.com/goods/index/id/10176<br>Anti-GST monoclonal antibody (CWBIO, CW0084), https://www.cwbio.com/goods/index/id/10104<br>Anti-beta-Actin monoclonal antibody (CWBIO, CW0264), https://www.cwbio.com/goods/index/id/10174

Anti-phosphoserine polyclonal antibody (Millipore, AB1603), https://www.merckmillipore.com/CN/zh/product/Anti-Phosphoserine-Antibody,MM_NF-AB1603
